# Plasticity in Adult Mouse Visual Cortex Following Optic Nerve Injury

**DOI:** 10.1093/cercor/bhy347

**Published:** 2019-01-21

**Authors:** Asta Vasalauskaite, James E Morgan, Frank Sengpiel

**Affiliations:** 1 School of Biosciences, Cardiff University, Museum Avenue, Cardiff, UK; 2 School of Optometry & Vision Sciences, Cardiff University, Maindy Road, Cardiff, UK; 3 Neuroscience & Mental Health Research Institute, Cardiff University, Maindy Road, Cardiff, UK

**Keywords:** axonal injury, imaging, mouse, ocular dominance plasticity, primary visual cortex

## Abstract

Optic nerve (ON) injury is an established model of axonal injury which results in retrograde degeneration and death of retinal ganglion cells as well anterograde loss of transmission and Wallerian degeneration of the injured axons. While the local impact of ON crush has been extensively documented we know comparatively little about the functional changes that occur in higher visual structures such as primary visual cortex (V1). We explored the extent of adult cortical plasticity using ON crush in aged mice. V1 function of the contralateral hemisphere was assessed longitudinally by intrinsic signal imaging and 2-photon calcium imaging before and after ON crush. Functional imaging demonstrated an immediate shift in V1 ocular dominance towards the ipsilateral, intact eye, due to the expected almost complete loss of responses to contralateral eye stimulation. Surprisingly, within 2 weeks we observed a delayed increase in ipsilateral eye responses. Additionally, spontaneous activity in V1 was reduced, similar to the lesion projection zone after retinal lesions. The observed changes in V1 activity indicate that severe ON injury in adulthood evokes cortical plasticity not only cross-modally but also within the visual cortex; this plasticity may be best compared with that seen after retinal lesions.

## Introduction

Since the seminal work by Hubel and Wiesel in the 1960s experience dependent plasticity in the primary visual cortex has been one of the most widely studied paradigms of learning processes in the brain. Monocular deprivation (MD) by eyelid suture remains the most commonly employed experiential modification, and its application has identified a critical period early in postnatal development when abnormal visual experience may affect the balance of inputs from the 2 eyes (Hubel and Wiesel 1970). MD results in the shrinkage of geniculocortical afferents from the deprived eye (Shatz and Stryker 1978; Antonini et al. 1999), a shift in ocular dominance of neuronal responses to the nondeprived eye (Wiesel and Hubel 1963; Olson and Freeman 1975), and a loss of vision (amblyopia) in the deprived eye (Dews and Wiesel 1970; Prusky and Douglas 2003). MD imposed after the critical period (which lasts up to about 7 years in human, 6 months in cats and 5 weeks in mice) has relatively little permanent effect, while plastic changes triggered during the critical period tend to be irreversible later in life. However, more recent research in rodents has questioned the rigidity of this critical period since numerous experimental manipulations have been shown to restore plasticity beyond the end of the critical period by removing “brakes on plasticity” (Bavelier et al. 2010; Takesian and Hensch 2013; Sengpiel 2014).

Another intervention that has been shown to cause compensatory plastic changes in the visual cortex both in juvenile and adult animals is deafferentation, obtained by either retinal lesions or monocular enucleation (ME). Spatially restricted retinal lesions matched in the 2 eyes cause initial silencing of the cortical lesion projection zone (LPZ), resulting in a scotoma. But cortical neurons that previously had receptive fields within the retinal lesion rapidly acquire new receptive fields adjacent to the lesion (Kaas et al. 1990), resulting in perceptual filling-in (De Weerd et al. 1995). The reorganization of the retinotopic map in the region of cortex representing the lesion (Gilbert and Wiesel 1992; Darian-Smith and Gilbert 1994), is underpinned by both short- and long-term physiological changes, with a peak of hyperactivity moving from the border into the hypoactive LPZ and forming the leading edge of a functional reconnection process during which cells inside the LPZ develop ectopic receptive fields and regain orientation selectivity (Giannikopoulos and Eysel 2006). This process is accompanied by extensive structural changes involving both excitatory and inhibitory neurons (Keck et al. 2008, 2011).

Deafferentation by ME induces striking plasticity which has been studied extensively in primates, both prenatally (Rakic 1976, 1981) and postnatally (Hubel et al. 1977), in each case resulting in compensatory reorganization of visual pathways. More recent studies in rodents have shown reorganization in both visual and nonvisual pathways (reviewed by Nys, Scheyltjens, et al. 2015). While ME early in life triggers both subcortical and cortical mechanisms of plasticity primarily within the visual system (Nys, Scheyltjens, et al. 2015), after ME later in life plasticity within the visual system is less pronounced and cross-modal plasticity based on changes in the corticocortical network is observed (Nys et al. 2014; Nys, Smolders et al. 2015).

Optic nerve (ON) injury which plays a major role in glaucoma represents a less drastic disruption to the retinocortical input than enucleation. ON injury is an established model of axonal injury which results in anterograde (Wallerian) degeneration of the distal axon. Ultrastructural changes indicating degeneration of the ON terminals in the lateral geniculate nucleus (LGN) are observed within 24 h, and signal transmission through degenerating synapses deteriorates rapidly, ceasing completely within 96 h (Eysel et al. 1974). This is followed by a decrease in neuronal some size in the LGN within a week; a decline in neuronal cell number and an increase in apoptotic cells is observed in V1 after 1 month (You et al. 2012). ON injury also results in progressive retinal ganglion cell death (Allcutt et al. 1984), through apoptosis, mediated in part by inflammatory processes in the retina (Bien et al. 1999). Adult mice are more susceptible to RGC loss after an ON crush than young mice, due to an increase in accompanying astrocyte death (Wang et al. 2007). However, little is known about how visual responses in downstream areas such as primary visual cortex (V1) are affected in either the short or the long term, apart from a single study employing partial ON crush and a metabolic activity mapping technique (Macharadze et al. 2012). Here, we studied responses in V1 of approximately 1-year-old mice over a period of 2–4 weeks following unilateral ON crush, using optical imaging of intrinsic signals and 2-photon calcium imaging.

## Materials and Methods

### Animals

All procedures were performed in accordance with the UK Animals (Scientific Procedures) Act 1986 and the European Commission directive 2010/63/EU. Mice were housed in groups in standard laboratory cages with food and water provided ad libitum and maintained on cycles of 14 h of light, and 10 h of darkness at 21 °C. Experiments were carried out on both male and female C57BL/6 mice aged 7 months and older (332 days ± 12 days, mean ± sem). This age range was chosen to represent mature adulthood, well beyond the period of developmental plasticity but before the onset of age-related degenerative processes in the visual system. Animals were housed in groups of 2–4 separated by gender.

### ON Crush

The ON crush procedure followed (Templeton and Geisert 2012) and was performed under general anesthesia. In brief, the ON was clamped for 3 s with curved self-closing forceps (#N7 Cross Action, Dumont) and the eye rotated back to its normal position. The retina was inspected with an ophthalmoscope, to confirm an intact retinal circulation in the ON-crushed eye. Ophthalmic ointment (1% Chloramphenicol) was applied to the eye and eyelid.

### Chronic Window Implantation Surgery

Anesthesia was induced using 4% isoflurane in oxygen, with 0.5 L/min flow rate, in an induction chamber. The NSAID Metacam (s.c., 1 mg/kg,) and dexamethasone (2 mg/kg, i.m.) were injected. The anesthesia level was maintained with 2–2.5% isoflurane in 0.3 L/min oxygen. A custom stainless steel headpost was attached to the skull using cyanoacrylate glue (3 M Vetbond) and dental acrylic (C&B-Metabond). A round (3 mm) craniotomy was made over V1, centered on the binocular portion V1b (stereotaxic coordinates 0.4 mm anterior and 3.2 mm lateral of lambda), and the skull flap completely removed and replaced with a glass window. The glass window was produced by gluing a small round (3 mm) of glass, to a 6 mm round cover-glass, using UV curable adhesive (NOA 61, Norland products). The edges of the glass were secured to the skull using the same cyanoacrylate glue and dental acrylic used to secure the headpost.

### Viral Delivery

At stereotaxic coordinates 0.4 mm anterior and 3.2 mm lateral of lambda 100–200 nL of a solution containing viral calcium indicator GCaMP6s (AAV1.Syn.GCaMP6s.WPRE.SV40) or mRuby-Gcamp6s (AAV1.hSyn1.mRuby2.GSG.P2A.GCamP6s.WPRE.SV40) (virus titers of 10^12^ genomes/mL, University of Pennsylvania Vector Core) was injected slowly at the rate of 20 nL/min into V1b of the right hemisphere at a depth of 200–300 μm below dura. The injection was performed using a 5 μL syringe (Hamilton) attached to an UltraMicro Pump with Micro4 controller (WPI, USA). The syringe was adapted with RN compression fittings (Hamilton) in order to accommodate custom pulled glass pipettes. Experiments commenced 3 weeks later when virus (and GCaMP6s) expression had reached its peak.

### Optical Imaging of Intrinsic Signals

Intrinsic signal imaging was performed on V1 contralateral to the eye that had received the ON crush using methods previously described (Erchova et al. 2017). Briefly, V1 was imaged under anesthesia using 0.8% isoflurane in O_2_ at 0.3 L/min, supplemented with chlorprothixene (i.m., 25 μg) sedation. Computer-controlled shutters were used to present stimuli to one eye at a time. Visual stimuli consisted of a bar of 40° in length and 4° width drifting upwards at a rate of 0.125 Hz, presented in the center of the visual field (the binocular zone) of the mouse at a distance of 14 cm. The stimulus was presented to each eye 6 times. Intrinsic signal images were captured using an Imager 3001 (Optical Imaging). Data analysis was performed using custom written software in MATLAB. For each pixel in the imaged region, the phase and amplitude of the optical signal at 0.125 Hz were calculated using FFT (Kalatsky and Stryker 2003). Response magnitudes are presented as Δ*R*/*R* values where *R* is light reflected. An ocular dominance index (ODI) was calculated on a pixel-by-pixel basis according to the following formula:

$$ODI=\frac{Contralateral-Ipsilateral}{Contralateral+Ipsilateral}$$

### Two-Photon Calcium Imaging

Two-photon imaging was performed under light anesthesia using 0.7–0.8% isoflurane in O_2_ at 0.3 L/min, supplemented with chlorprothixene (i.m., 25 μg) sedation. Imaging was performed on a custom built 2 P microscope (MOM, Sutter Instruments) equipped with Ti:Sapphire laser (MaiTai DeepSee, Newport SpectraPhysics) using a × 20 Olympus (1.00 NA, 2.0 mm WD, N20X-PFH) water immersion objective. The laser was tuned to 940 nm and the power maintained in the range of 25–35 mW. Image frames of 256 × 256 pixels representing an area of 270 μm × 270 μm located in binocular V1 (V1b), at approximate stereotaxic coordinates 0.4 mm anterior and 3.2 mm lateral of lambda, were collected at depths of 180–250 μm at 3.4 fps (ScanImage r3.6) while the mouse was presented with visual stimuli shown to one eye at a time. 16 directions of motion in 22.5° steps of either a 4° wide and 40° long white bar on a black background drifting at 0.125 Hz or 40° × 40° gratings of 0.04 cycles/deg (cpd) spatial frequency and 2 cycles/s temporal frequency were presented at a viewing distance of 20 cm. This was followed by either presentation of the black background alone (blank control for the bar stimuli) or gray screen (blank control for the gratings). Stimuli were presented for 8 s and the interstimulus interval was 7 s long. Each stimulus was presented 4 times to each eye for each imaged area. Spontaneous activity was recorded separately, either before or after recording visually evoked responses. The spontaneous activity in the region of interest was collected continuously for 10 min with the mouse facing a dark screen and eye shutters open.

### Data Analysis

Two-photon calcium image analysis was performed using custom software written in MATLAB (Ranson 2017). All images collected from the same area were automatically aligned, and corrected for in-plane motion using a correlation-based subpixel registration. Cell masks were manually assigned based on average calcium signals. Cellular fluorescence time courses were extracted by averaging the pixels within each cell mask, traces were then low-pass filtered (0.8 Hz) and adjusted by subtracting the corresponding neuropil signal. Response was calculated for each trial as follows:

$$\Delta R/R=\;\frac{\;R-R_{0}}{R_{0}}$$

where *R* is fluorescence signal and *R*_0_ is a mean baseline fluorescence signal over a 2 s period immediately before the start of stimulus presentation. Visual responses were taken as the mean response over the full duration to each stimulus presentation during individual trials. Neurons were defined as visually responsive if a significant difference was observed in any of 16 directions of 4 trial means (*P* < 0.05, one-way ANOVA). Orientation tuning curves were derived from a Gaussian curve fit to the visual responses to each of the 16 stimuli.

The ODI of individual neurons was calculated as follows:

$$ODI=\frac{R_{max}Contralateral-R_{max}Ipsilateral}{R_{max}Contralateral+\;R_{max}Ipsilateral}$$

*R_max_* represents mean response magnitudes for each eye to drifting bar or grating stimuli at the preferred direction.

The spontaneous activity of each cell was extracted per trial as a single fluorescent trace from averaged pixels within each cell mask (see above). The median of the full trace was calculated and values that fell between 0.9 and 0.1 quantiles were used as a baseline trace to calculate a mean and standard deviation (*σ*). Events that were 2 × *σ* above the baseline with at least 1/3 higher peak prominence relative to neighboring peaks were defined as significant. Activity classification was based on previously described spontaneous activity patterns in adult mouse V1 (Grienberger et al. 2012), “silent” or hypoactive neurons were defined as neurons with < 0.25 events/min, “normal” as 0.25–4 events/min and “hyperactive” > 4 events/min.

To examine the synchronicity of detected spontaneous events a binary matrix representing the events per frame in a single field of view was constructed. The events in each frame were added resulting in a number of coactive neurons per frame. The events in the original binary matrix were then reshuffled 1000 times and from the constructed histogram, a threshold of coactive cells at *P* < 0.01 value was determined (Fang and Yuste 2017). Events in frames that contained a higher number of coactive neurons than the threshold were defined as synchronous events.

### Histology

To validate our experimental model we collected retinas from mice at 2 (*n* = 3), 7 (*n* = 4), 14 (*n* = 4), and 28 days (*n* = 4) after ON crush. Retinas were dissected as retinal wholemounts and then stained with the nuclear dye Hoechst. This simple stain was chosen over more RGC specific immunohistochemistry since we primarily required confirmation that the ON crush was effective across the whole extent of the retina. Four areas (0.01 mm^2^ per retina) were sampled 700 µm from the ON head. Only stained cells in the ganglion cell layer (GCL) were counted.

### Experimental Design and Statistical Analysis

Statistical analyses were performed using either SPSS v. 20.0 (IBM Corp, USA) or Prism 7 (GraphPad Software, USA). Data are reported as median and inter quartile ranges (IQR) or mean ± standard error of mean (sem). Data were first tested for normality and then analyzed appropriately using either parametric (*t*-test, one way ANOVA) or nonparametric (Mann–Whitney *U* test, Kruskal–Wallis (KW) test with post hoc Dunn’s test) tests. Distribution data were tested by using a chi square test. Detailed descriptions of statistical tests used, significance values, and animal and sample numbers are given in figure legends.

## Results

### Retinal Ganglion Cell Degeneration

**Figure 1.**
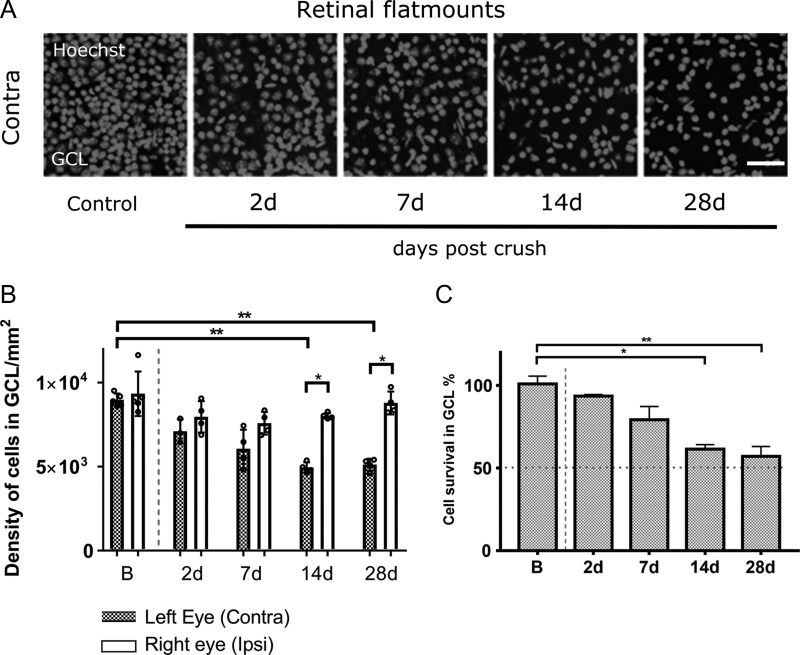
Classification of cell loss in ganglion cell layer (GCL) of retina after unilateral ON crush. (*A*) Confocal images showing Hoechst stained cell nuclei in GCL of flat mounted retinal preparations from contralateral eyes after ON crush. Scale bar, 100 μm. (*B*) Density of Hoechst positive cells in GCL of contralateral and ipsilateral eyes. Bars represent median cell density and small circles are individual density values (contra B vs. contra 14 d; contra B vs. contra 28 d; **P < 0.01; Kruskal–Wallis test with Dunn’s post hoc test; contra 14 d vs. ipsi 14 d; contra 28 d vs. ipsi 28 d; *P < 0.05; Mann–Whitney test). (*C*) Quantification of surviving cells in GCL of retina, represented as percentage of surviving cells in GCL in the injured eye (contra), compared with the intact fellow eye (ipsi) (B vs. 14 d, *P < 0.05; B vs. 28 d; **P < 0.01; Kruskal–Wallis test with Dunn’s post hoc test). (*B*, *C*) B, 5 mice; 2 d, 3 mice; 7 d, 4 mice; 14 d, 4 mice; 28 d, 4 mice; Data are presented as medians and IQR.

ON crush caused a marked and reproducible reduction in retinal ganglion cell density, as assessed histologically, which started within 48 h (Fig. 1*A*). The most marked change occurred within the first 14 days when a significant decrease in density of RGCs was observed in ON-crushed eyes compared with control eyes (KW test, *P* = 0.0027) (Fig. 1*B*). When we expressed cell density in the ON-crushed eye relative to the density in the fellow eye, we observed a gradual decrease from 93% survival at 2 days to 57% at 28 days (Fig. 1*C*). Considering that over 50% of cells in the RGC layer labeled by our nuclear stain are displaced amacrine cells (Jeon et al. 1998) the extent of RGC death was near-total, indicating that the ON crush caused axons to be lost across the entire cross-section of ON.

### Optical Imaging

**Figure 2.**
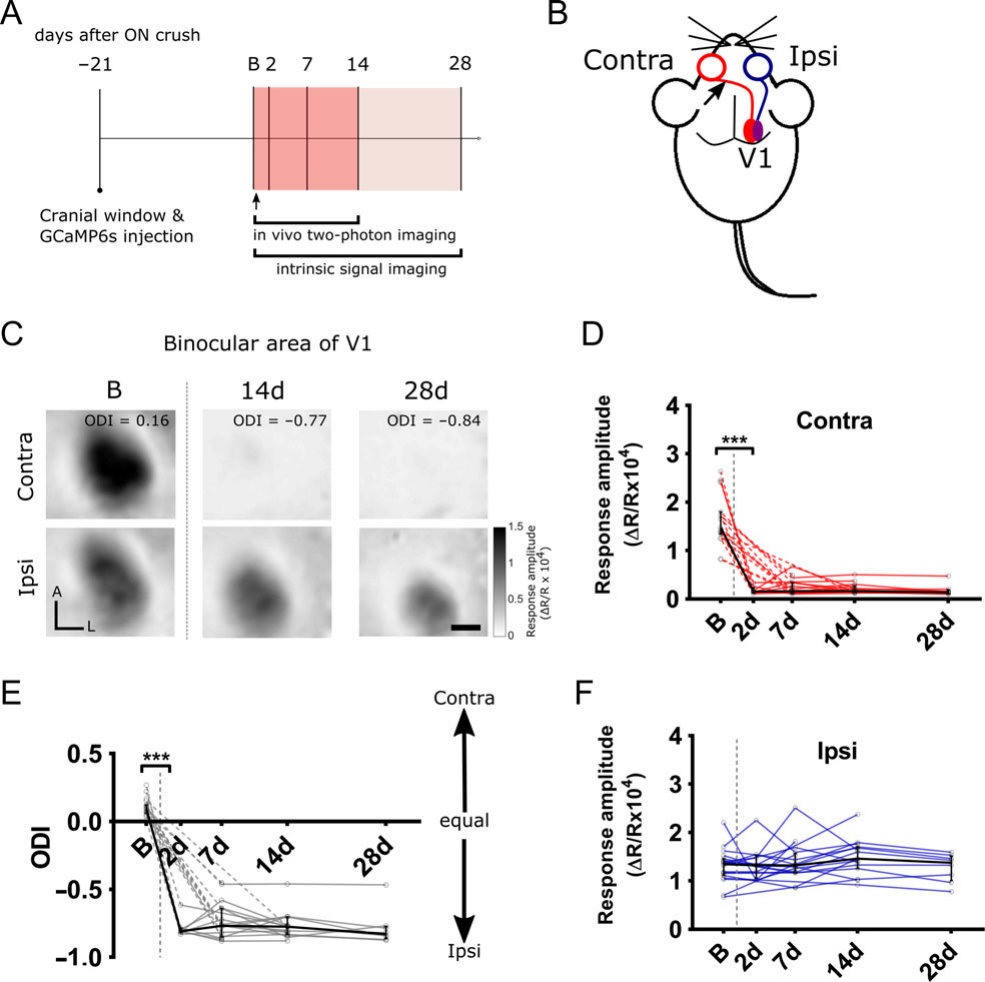
Cortical V1b responses measured by intrinsic signal imaging after unilateral ON crush. (*A*) Experimental timeline to examine the cortical responses after unilateral ON crush. B—baseline (control) session, arrow indicates the time point of ON crush on contralateral eye. (*B*) Schematic representation of the experimental design. Arrow indicates ON crush site on contralateral side to the imaged V1 hemisphere. The binocular portion of V1 (V1b) is indicated by purple color. (*C*) Examples of intrinsic signal imaging (ISI) responses to the visual stimuli presentation to contralateral (top panel) and ipsilateral (bottom panel) eye in the binocular area of V1 of the same mouse. Panels show V1b responses to either eye obtained at baseline (left), 14 (middle) and 28 days (right) after unilateral ON crush was performed on the contralateral eye. A—anterior, L—lateral, ODI—ocular dominance index. Scale bar, 0.5 mm. (*D*,* E*) V1b responses to contralateral and ipsilateral eye stimulation. Red and blue lines are individual mouse response values. (Contra: B vs. all post crush time points, ***P < 0.001; Ipsi: B vs. postcrush time points, n.s.). (*F*) Ocular dominance index (ODI) values calculated from V1b responses to contralateral and ipsilateral eye after unilateral ON crush. gray lines are individual mouse ODI values (B vs. all post crush time points, ***P < 0.001). (*D*–*F*) Dashed lines connect missing values. Black line is a median and IQR, vertical gray dashed line is the time point of ON crush (B, 19 mice; 2 d, 8 mice; 7 d, 17 mice; 14 d, 15 mice; 28 d, 8 mice; Kruskal–Wallis test with Dunn’s post hoc test).

For functional assessments, 19 mice were imaged immediately before the unilateral ON crush. These were then imaged again post ON crush, at 2 days (*n* = 8), 7 days (*n* = 17), 14 days (*n* = 15) and 28 days (*n* = 8). The timeline for the experiment is summarized in Figure 2*A*. Typical activity patterns in V1 for contralateral (ON-crushed) eye and ipsilateral eye stimulation before the crush as well as 14 and 28 days later are shown in Figure 2*C*. Figure 2*D*–*F* shows the changes in ODI as well as in the absolute magnitude of responses through the 2 eyes over the time course of the experiment. By comparison, responses through each eye as well as ODI remained stable in 15 age-matched control animals that were imaged twice at an interval of 2 weeks (Suppl. Fig. S1).

In contrast with the more gradual decline in the number of live retinal ganglion cells (Fig. 1), the loss of responses elicited in V1 through stimulation of the ON-crushed eye, contralateral to the imaged hemisphere, was dramatic and complete within 2 days, indicating that anterograde signal transmission was disrupted across the entire ON. There was virtually no detectable signal in any of the animals imaged 2 days after the crush, with the response dropping from 1.64 ± 0.12 (mean ± sem; Δ*R*/*R* × 10^4^) before the crush to 0.178 ± 0.028 after 2 days. The responses through the ON-crushed eye remained negligible throughout the follow-up period, and were at 0.179 ± 0.043 (mean ± sem; Δ*R*/*R* × 10^4^) after 28 days. Accordingly, the ODI dropped from 0.103 ± 0.014 (mean ± sem) just before the ON crush to −0.78 ± 0.025 after 2 days, and did not change significantly thereafter, averaging −0.78 ± 0.047 after 28 days. The magnitude of responses elicited through the ipsilateral eye was 1.31 ± 0.080 before the ON crush in the other eye; it rose slightly but not significantly to 1.49 ± 0.096 (mean ± sem) over 14 days post ON crush and decreased slightly but not significantly to 1.28 ± 0.10 after 28 days. The latter decrease may have been caused by a slightly reduced optical quality of the cranial window. Taken together, optical imaging of intrinsic signals did not show any significant change in the responses elicited through the intact eye, presumably because the intersession variability of signals did not allow us to reliably detect relatively small changes.

### Calcium Imaging of Neuronal Responses

**Figure 3.**
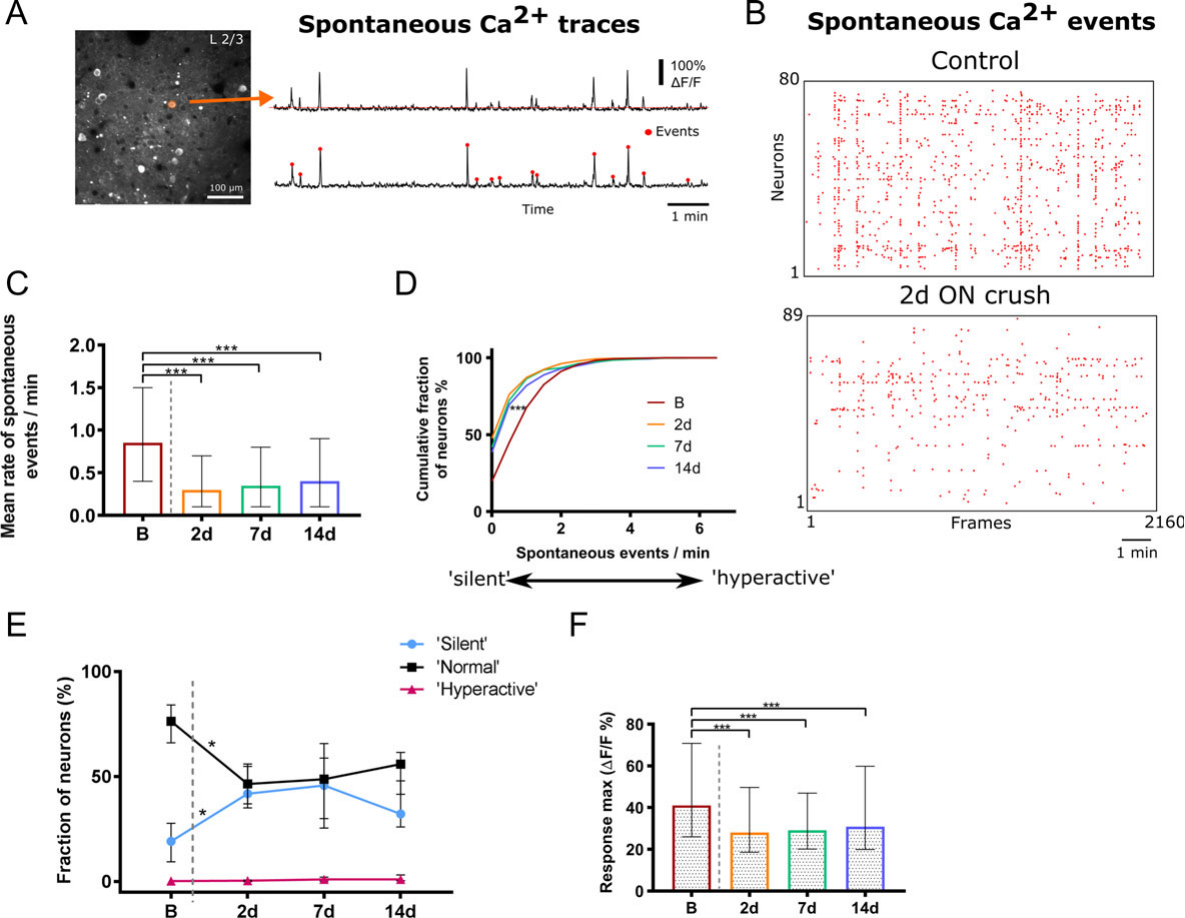
Spontaneous activity in L2/3 of V1b after unilateral ON crush. (*A*) In vivo 2-photon microscope image showing a typical field of view of GCaMP6s labeled neurons in L2/3 of V1b (left). Orange circle is indicating a cell mask that was used to extract spontaneous Ca^2+^ fluorescence traces shown on the right (top trace). Significantly spontaneous events (red circles) were then detected from 10 min recording (bottom trace) as described in methods section. (*B*) Raster plot of detected spontaneous Ca^2+^ events of all identified neurons in a single field of view before (top) and 2d after (bottom) unilateral ON crush in the same animal over 10 min of recording. (*C*) The mean rate of spontaneous events and (*D*) normalized frequency distribution of all detected spontaneous events after the ON crush. The rate is expressed as spontaneous events per neuron per minute (B vs. all post crush time points, ***P < 0.001). (*E*) Quantification of mean fractions of “silent” (0–0.25 events/min), “normal” (0.25–4 events/min) and “hyperactive” (>4 events/min) among all detected spontaneous events, before and after ON crush (“silent” B vs. “silent” 2 d, *P = 0.015; “normal” B vs. “normal” 2 d, *P = 0.013). (*F*) Maximum response magnitudes of spontaneous events after the ON crush (B vs. all post crush time points, ***P < 0.001). (*C*–*F*) B, *n* = 1040 neurons, 6 mice; 2 d, *n* = 797 neurons, 5 mice; 7 d *n* = 448 neurons, 4 mice; 14 d *n* = 686 neurons, 4 mice; (*C*, *E*, *F*) Kruskal–Wallis test with Dunn’s post hoc test. Data are presented as median and IQR.

In order to obtain a more detailed picture of the cortical changes triggered by unilateral ON crush we carried out chronic 2-photon calcium imaging of layers 2/3 of V1. We observed that spontaneous activity, as measured by the number of calcium events per minute (Fig. 3*A*), was reduced following contralateral ON crush. Typical detected event plots from a single field of view of one animal obtained immediately before and 7 days after the ON crush are shown in Figure 3*B*. The mean number of events/min (per neuron) decreased significantly from 0.85 (0.4–1.5) (median and IQR) before the crush to 0.3 (0.1–0.7) 2 days after the crush (*P* < 0.001, KW test), and remained at a similar level by 14 days after the crush (Fig. 3*C*,*D*). When we classified neurons according to their level of spontaneous activity as previously described (Busche et al. 2008; Grienberger et al. 2012), we found that silent or hypoactive cells (<0.25 events/min) were significantly rarer (*P* = 0.0154, KW test) in mice with intact ON than at any time point after the ON crush. In contrast, neurons with normal levels of spontaneous activity (0.25–4 events/min) were more frequent before the ON crush, while numbers of hyperactive cells (>4 events/min) remained unchanged before and after the ON crush (Fig. 3*E*). The frequency distribution was significantly different after compared with before the ON crush (*x*^2^ > 96, *P* < 0.001, Chi square test). In addition, as shown in Figure 3*F* the maximum responses (Δ*F*/*F*) of spontaneous events were found to be significantly reduced from 41.13 (26.05–70.8) (median and IQR) before the crush to 28.06 (18.66–49.68) 2 days and up to 14 days after (all time points *P* < 0.001, KW test). Age-matched control animals, which were imaged twice 14 days apart, did not exhibit a significant change in either mean rate of spontaneous events or the relative frequency of neurons exhibiting hypo-, normal, and hyperactivity (Suppl. Fig. S2).

**Figure 4.**
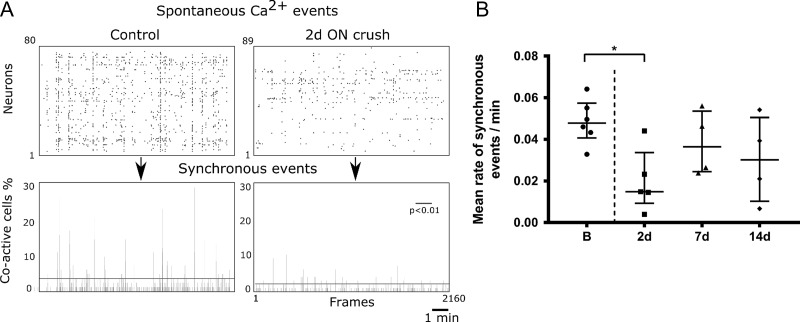
Synchronous spontaneous activity in L2/3 of V1b after ON crush. (*A*) Examples of raster plots of detected spontaneous events (top panels) and sum of events per frame in single field of view (bottom panels) over 10 min of recording. Synchronous events are defined as the events above the horizontal line (bottom panels; *P* < 0.01; random permutation test). (*B*) Mean rate of synchronous events before and after ON crush. Synchronous events are expressed per neuron per minute (B vs. 2 d, *P* = 0.0294). Small circles are mean values of individual mice, error bars are medians and IQR (B, 6 mice; 2 d, 5 mice; 7 d, 4 mice; 14 d, 4 mice; Kruskal Wallis test with Dunn’s multiple test).

**Figure 5.**
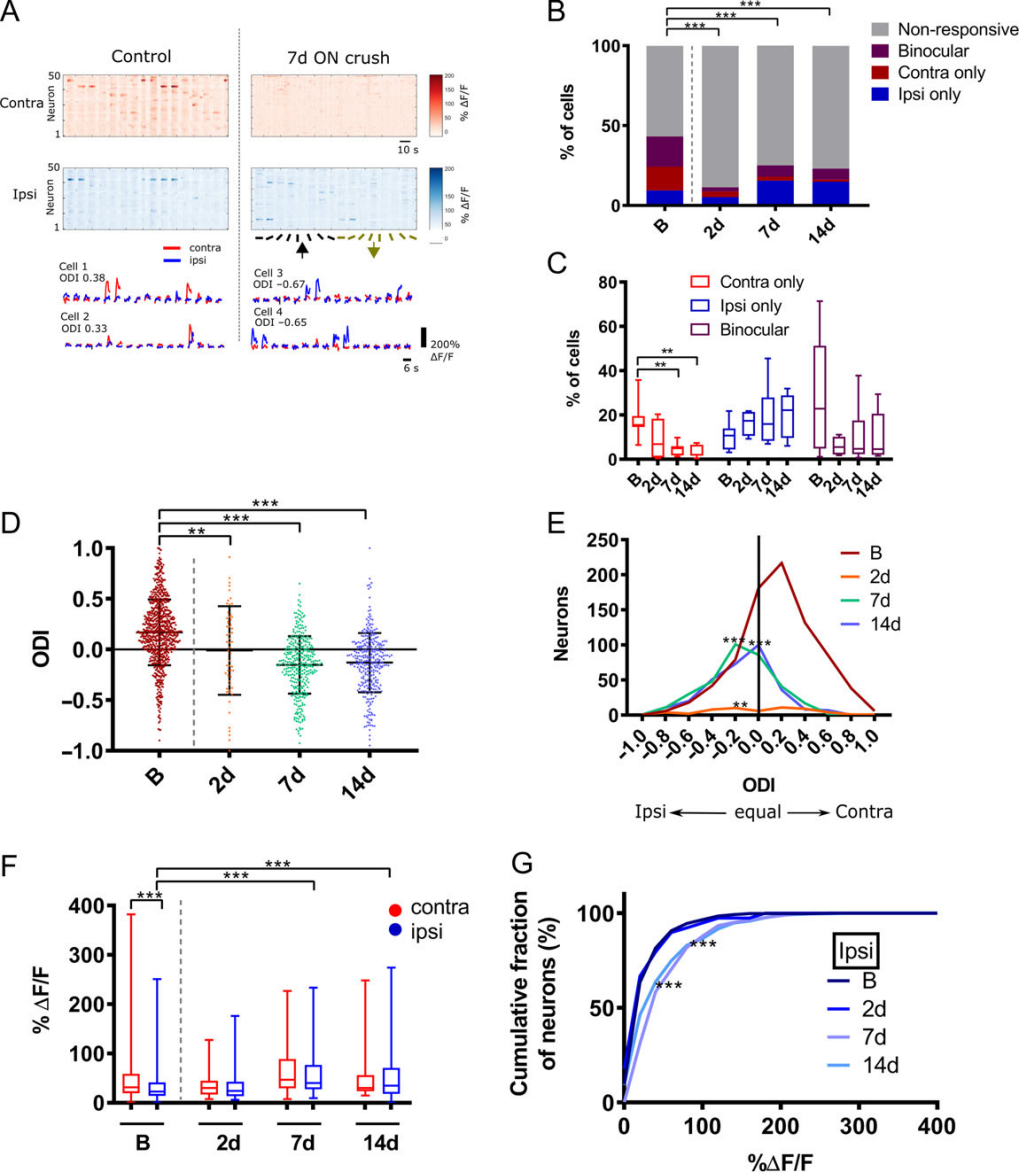
Visually evoked responses in L2/3 of V1b neurons after unilateral ON crush. (*A*) Top—responses as fluorescence changes of 50 neurons from a single field of view to contralateral and ipsilateral visual stimuli of 16 directions; the same field of view was imaged before (control) and after ON crush. Bottom—examples of mean Ca^2+^ traces of 4 individual neurons to contralateral and ipsilateral stimulation. Cells 1 and 2 were recorded before and cells 3 and 4 after ON crush. Left panel column shows responses at baseline (control) and right panel data from the same animal 7 days after ON crush. ODI, ocular dominance index; red, contralateral; blue, ipsilateral. (*B*) Quantification of overall neuronal responsiveness (nonresponsive, binocular only, contralateral only, ipsilateral only) to visual stimuli after the ON crush (B, *n* = 1856 neurons, 11 mice; 2 d, *n* = 499 neurons, 4 mice; 7d, *n* = 1344 neurons, 8 mice; 14d, *n* = 1337 neurons, 8 mice; B vs. all post crush time points, ***P < 0.001, Chi-square test). (*C*) The distribution of each of the visual responsive fractions after the ON crush. Data are presented as median, hinges—IQR, whiskers—min/max (B, 11 mice; 2 d, 4 mice; 7 d, 8 mice; 14 d, 8 mice; contralateral fractions at B vs. 7 d, **P = 0.0037, B vs. 14 d **P = 0.0013). (*D*) Bee swarm plot (median and IQR) and (*E*) frequency distribution of ODI values of all visually responsive neurons in L2/3 V1b neurons after the ON crush (B vs. 2 d, **P = 0.0017, B vs. 7 d and B vs. 14 d, ***P < 0.001). (*C*–*E*) Kruskal–Wallis test with Dunn’s post hoc test. (*F*) Mean response amplitudes of contralateral and ipsilateral responsive neurons after the ON crush and (*G*) cumulative distribution of ipsilateral response amplitudes (contra B vs. 7d, ***P < 0.001; ipsi B vs. 7 d and B vs. 14 d, ***P < 0.001; Kruskal–Wallis test with Dunn’s post hoc test; B contra vs. B ipsi, ***P < 0.001; Mann Whitney test). (*F*) Data are presented as box plots with median (hinges—IQR, whiskers—min/max). (*F*,* G*) B, contra *n* = 629 neurons, ipsi *n* = 528 neurons, 11 mice; 2d, contra *n* = 30 neurons, ipsi *n* = 39 neurons, 4 mice; 7d, contra *n* = 127 neurons, ipsi *n* = 304 neurons, 8 mice; 14d, contra *n* = 108 neurons, ipsi *n* = 289 neurons, 8 mice.

We further analyzed whether ON crush affected the degree of synchronicity of spontaneous network events (Fig. 4). Examples of raster plots of detected spontaneous events in a control animal versus 2 d post ON crush confirm the reduced number of events following ON crush (Fig. 4*A*). Synchronous events were defined as cells being coactive within a single image frame of 300 ms, thresholded at a *P* < 0.01 significance level (see Materials and Methods) in order to account for different absolute activity levels. The mean rate of synchronous events per minute was significantly lower 2 d post ON crush compared with baseline (Fig. 4*B*; Kruskal Wallis test with Dunn’s multiple test) and recovered to near baseline level by 7 d post ON crush. During presentation of visual stimuli 43.3% of all imaged neurons were visually responsive before the ON crush. An example field of view (top) and a single cell’s (bottom) responses through each eye to visual stimulation is displayed in Figure 5*A*. The proportion of visually responsive cells dropped to 25% 1 week after the crush and 23% after 2 weeks. Among the visually responsive neurons 9.4% responded to stimulation of the ipsilateral (intact) eye before the crush, rising to 15.0% 2 weeks after, while the proportion responding to stimulation of the contralateral (ON-crushed) eye fell from 14.8% before the crush to 2.5% after 1 week and 1.3% after 2 weeks (Fig. 5*B*,*C*). In parallel, the proportion of binocularly responsive neurons decreased from 19.0% to 7.0% after 1 and 6.7% after 2 weeks (*x*^2^ > 179, *P* < 0.001, Chi square test). Accordingly, the ODI of all visually responsive neurons was reduced significantly (*P* = 0.0017, KW test), from 0.16 (−0.01–0.37) before the ON crush to −0.13 (−0.32-0.03) one week later and −0.11 (−0.31 to 0.05) 2 weeks later (Fig. 5*D*,*E*). We next analyzed the magnitude of responses (Δ*F*/*F*) through each eye individually (Fig. 5*F*). We observed no change in the mean magnitude of the few remaining contralateral eye responses after the crush. However, we observed a significant increase in the magnitude (% Δ*F*/*F*) of ipsilateral eye responses in the week after the ON crush (Fig. 5*G*), from 22.3 (13.7–41.5) to 40.05 (27.5–76.9) and 34.4 (18.2–70.6) (*P* < 0.001, KW test) during the following week. In addition, the contralateral compared with ipsilateral responses were significantly higher before the crush (*P* < 0.001, Mann Whitney test), but were not significantly different at any time point after the crush.

## Discussion

The most striking finding in this study is that the responses in V1 through the intact eye increased 1–2 weeks after ON crush. These findings indicate that unilateral severe injury to the ON in adulthood may invoke plasticity mechanisms more typically seen during the critical period. This compensatory plasticity has not been demonstrated before at the single-cell level, for either ON crush or ME paradigms. There are, however, parallels with compensatory plasticity after focal retinal lesions.

While retrograde degeneration of retinal ganglion cells progressed over a period of 2 weeks after ON crush, disruption of visual signal relay to the primary visual cortex was immediate and permanent, in line with previous reports that ultrastructurally, degeneration of ON terminals in the LGN occurs within 24 h, and signal transmission through retinogeniculate synapses ceases completely within 96 h (Eysel et al. 1974). While there was a significant increase in intact-eye visual responses at 1 and 2 weeks after ON crush, the rate of spontaneous activity was reduced immediately after the ON crush and remained reduced, primarily because the proportion of hypoactive neurons was increased. A similar time course of postlesion plasticity has also been reported in a recent study that employed unilateral focal retinal lesions in adult mice (Smolders et al. 2016). Cortical activity measured in terms of immediate-early gene expression was strongly reduced in the LPZ 2 days postlesion but showed substantial recovery after one and full recovery after 3 weeks. Notably, this recovery is restricted to the visual cortex; none is seen in the superior colliculus (Smolders et al. 2016).

### Mechanism(s) of Plasticity

In order to understand the processes underpinning the observed adult plasticity following ON crush a comparison with the effects of retinal lesions offers important insights. A study on the lateral geniculate nucleus in cats showed that after monocular deafferentation increased inhibition is prevalent in the deafferented layer for several weeks but is later followed by increased excitation through the intact eye (Eysel 1979). After homonymous binocular retinal lesions Giannikopoulos and Eysel (2006) found that while the center of the LPZ is rendered hypoactive (like V1 contralateral to the ON crush in our study) a transient band of hyperactivity moves into the LPZ from the surrounding intact cortex over several weeks, preceding the functional reconnection process. Since we did not monitor responses of individual identified neurons over time, we cannot distinguish with certainty between increased responsiveness of neurons that were already dominated by the intact eye before the ON crush and newly acquired responsiveness to intact-eye stimulation of neurons that were previously dominated by the ON-crushed eye. However, the latter explanation seems more likely given that the proportion of cells responding to the ipsilateral (intact) eye increased 1 and 2 weeks post ON crush (Fig. 5*B*). Interestingly, a study that employed partial ON crush and a metabolic activity mapping technique found stronger recovery in the monocular zone; this also argues in favor of newly acquired responsiveness (Macharadze et al. 2012).

One candidate cellular mechanism for the response to deafferentation is homeostatic plasticity. Given the significantly reduced afferent activity in the hemisphere contralateral to the ON crush, homeostatic mechanisms such as synaptic scaling (Turrigiano et al. 1998) or increased intrinsic excitability (Desai et al. 1999) could increase responsivity of V1 neurons within a few days. For example, 2 or 3 days of total visual deprivation by dark exposure have been shown to cause a (reversible) increase in AMPA receptor-mediated miniature EPSC amplitude in layer 2/3 neurons (Goel and Lee 2007; Ranson et al. 2012). This homeostatic plasticity is known to depend on Tumor Necrosis Factor-α (TNF-α) (Kaneko et al. 2008); it is thought to involve multiplicative scaling which has been reported in cultured neurons after pharmacological activity blockade (Turrigiano et al. 1998) and in layer 2/3 pyramidal neurons in the visual cortex after dark exposure during the critical period, but not in adulthood (Goel and Lee 2007). Our finding that a greater proportion of neurons showed reduced spontaneous activity in contralateral V1 following ON crush while there was no evidence of cells exhibiting hyperactivity, indicates that homeostatic plasticity was not a factor. This appears to be consistent with the age of the animals in our study since homeostatic plasticity in response to MD is limited to the critical period (Mrsic-Flogel et al. 2007; Kaneko et al. 2008). Homeostatic plasticity mediated by synaptic scaling (Keck et al. 2013) as well as increases in spine size (Barnes et al. 2017) have been observed in adult mice but deafferentation of the visual cortex in those studies (unlike in ours) was complete, and the age of the animals was 60–120 days and therefore within the time window for adult visual cortex plasticity of standard-housed mice (Lehmann and Löwel 2008). Plasticity in animals older than 6 months has previously been described only after extensive environmental enrichment (Greifzu et al. 2014), which was not provided in our study. Similarly, cohousing of mice (as in our study) has been shown to promote adult plasticity (Balog et al. 2014), but animals in that study were less than 6 months old. However, one caveat regarding the comparison of those MD studies that only employed intrinsic signal imaging is the fact that we only observed upregulation of responses through the non-ON crushed with the more sensitive 2-photon calcium imaging; it can therefore not be ruled out that some nondeprived eye potentiation went undetected in those MD studies. The reduced synchronicity of spontaneous activity that we observed immediately following ON crush may have contributed to the lack of homeostatic plasticity since it has been shown that only those excitatory neurons homeostatically recover after ME whose activity correlates with that of other recovering excitatory neurons (Barnes et al. 2015).

Our earlier work on mechanisms of plasticity in mouse visual cortex (Ranson et al. 2012) suggested that adult plasticity is most likely to involve an LTP-type (Hebbian) process since it requires autophosphorylation of α-calcium/calmodulin-dependent protein kinase II. Our main finding of an increase in response through the intact eye appears to resemble the finding by (Frenkel and Bear 2004): monocular inactivation by intravitreal injection of tetrodotoxin promotes potentiation of responses driven by the noninjected eye. However, the mice in that study were within the critical period while ours were nearly 1 year old. The underlying mechanism is therefore likely to be different (Ranson et al. 2012). A recent study using a unilateral lesion of the infraorbital nerve to remove sensory input found that LTP was transiently restored in the spared layer 4 barrel cortex of adult mice (Chung et al. 2017), with a shift in NMDA receptor subunit composition to GluN2B at thalamocortical synapses (normally prevalent in early postnatal sensory cortex but replaced in development by GluN2A). Similar experience dependent changes in the expression of NMDA receptor subunits have previously been described for the rodent visual cortex as a consequence of a period of dark exposure (Quinlan et al. 1999; Philpot et al. 2001). They have been interpreted in the context of the Bienenstock–Cooper–Munro (BCM) theory which proposes a sliding threshold for LTP or LTD induction dependent on the time-averaged postsynaptic activity (Bienenstock et al. 1982; Philpot et al. 2003). It remains to be shown whether the intact-eye response potentiation we observed is NMDA receptor dependent.

### Comparison With ME and MD

While there are no previous accounts of the cortical consequences of ON crush, ME might be expected to be similar in terms of downstream effects. These depend significantly on timing (Nys et al. 2014): following ME, swift open-eye potentiation was observed in the binocular visual cortex of 45 days old mice but to a lesser extent in 120 days old mice. Our finding of intact-eye potentiation at nearly a year of age is therefore surprising. On the other hand, the later cross-modal reactivation of V1 by somatosensory inputs (as measured by immediate early gene expression) is much more robust after ME in adult mice than in juvenile ones (Nys et al. 2014). It would therefore be worth examining whether somatosensory responses are enhanced in the cortical hemisphere contralateral to the ON crush.

While one might intuitively expect greater plasticity in the visual cortex after ME or ON crush than after MD since the latter preserves afferent input from the affected eye, it has been argued that homosynaptic LTD of deprived-eye inputs should be stronger in the presence of decorrelated afferent activity (as is the case in MD) than in the absence of any afferent activity (as in ME or ON crush). This prediction is supported by the BCM theory (in which no activity results in no change of synaptic weights) and borne out by a study that showed a larger ocular dominance shift in monocularly deprived kittens than in animals which had retinal activity silenced in one eye by tetrodotoxin injections (Rittenhouse et al. 1999). Similarly, in juvenile mice monocular inactivation fails to induce deprived-eye response depression but promotes potentiation of responses driven by the fellow eye (Frenkel and Bear 2004), resembling the effects of MD in adult mice (Sawtell et al. 2003).

### Clinical Relevance of Adult Plasticity

ON trauma is clinically a distinct entity which has also been used, with caveats, as a model of other more commons optic neuropathies such as glaucoma. The common pathology is of selective retinal ganglion cell loss, and as such our data are relevant to consideration of the central effects of glaucoma-related vision loss. They complement a recent study into the central effects of induced monocular hypertension in adult mice which showed hypoactivity in contralateral V1 and V2 after 1 week but a return to normal activity levels after 4 weeks (Dekeyster et al. 2015). It is important to note that ON crush induces more rapid RGC loss compared with models of glaucoma driven by elevated intraocular pressure (Vidal-Sanz et al. 2017) so clinical extrapolations should be conservative. Interestingly, while the overall percentage of RGCs that survive ON crush long term is very small, the proportion of melanopsin-positive intrinsically photosensitive RGCs (ipRGCs) surviving up to 6 months post ON crush is around 40% (Nadal-Nicolas et al. 2015). Some ipRGCs project to the LGN (Dacey et al. 2005) and may therefore support image-forming vision (Ecker et al. 2010), in particular irradiance coding (Brown et al. 2010). This might help to explain the remaining cortical responses to stimulation of the ON-crushed eye in our study.

## Supplementary Material

Supplementary Data
