# Supplementary material for: Plasticity in Adult Mouse Visual Cortex Following Optic Nerve Injury
Source: Cereb Cortex. 2019 Jan 21;29(4):1767–77. doi: 10.1093/cercor/bhy347 (PMC6418869; doi:10.1093/cercor/bhy347)
Supplement: Supplementary Data [file bhy347_supplementary_materials.zip › bhy347_Plasticity_in_adult_mouse_visual_cortex_supp_legends.docx]

**Supplementary Figures**

Supplementary Figure 1

**Intrinsic signal imaging of control animals.**

**(A)** Examples of intrinsic signal imaging (ISI) responses of V1b to contralateral (top panel) and ipsilateral (bottom panel) eye stimulation in the same mouse over two baseline sessions. B1 – first baseline session, B2 – second baseline session, A - anterior, L – lateral, ODI – ocular dominance index. Scale bar, 0.5 mm.

**(B)** ODI values obtained from the first and second baseline imaging sessions.

**(C,D)** V1b response amplitudes to contralateral and ipsilateral eye stimulation,

**(B-D)** Grey, red, blue lines – values obtained from each mouse, black line – mean and standard deviation; 16 mice, n.s., paired t-test.

Supplementary Figure 2

**Spontaneous activity in L2/3 of V1b in control animals.**

**(A**) Mean rate and **(B)** normalised cumulative distribution of spontaneous events at two baseline sessions.

**(C)** Mean fractions and **(D)** summary of neuronal fractions of ‘silent’ (0-0.25 events/min), ‘normal’ (0.25-4 events/min) and ‘hyperactive’ (>4 events/min) neurons at two baseline sessions

**(E)** Maximum response magnitudes of spontaneous events at two baseline sessions.

**(A-C, E)** Data are presented as median and IQR (B1, n=525 neurons, 4 mice; B2, n=596 neurons, 4 mice, Mann Whitney test). **(D)** Chi square test.
