# Supplementary figures and images for: Plasticity in Adult Mouse Visual Cortex Following Optic Nerve Injury
Source: Cereb Cortex. 2019 Jan 21;29(4):1767–77. doi: 10.1093/cercor/bhy347 (PMC6418869; doi:10.1093/cercor/bhy347)

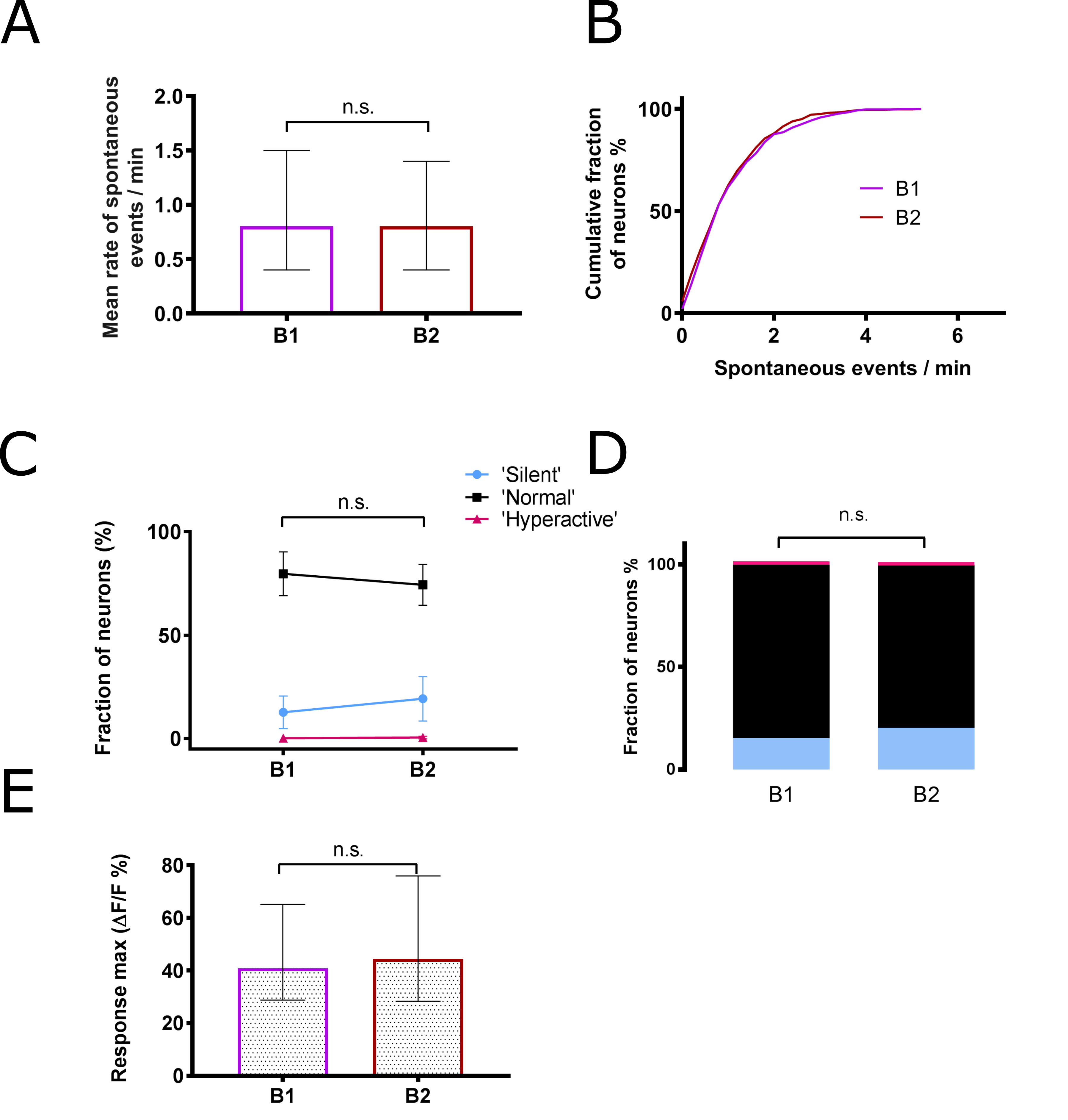

Supplement: Supplementary Data [file bhy347_supplementary_materials.zip › bhy347_supp2.tif]
